# Greenhouse gas emissions due to long-term data storage of CT with reformats and strategies for mitigation

**DOI:** 10.1007/s00330-025-12023-z

**Published:** 2025-09-30

**Authors:** Yifan Jia, Michael Deng, Rebecca Burger, Sarah Sheard, Kate Hanneman, Moran Drucker Iarovich, Evis Sala, Giacomo Avesani, Rowland O. Illing, Andrea G. Rockall

**Affiliations:** 1 Department of Medicine, Imperial College London, London, UK; 2 Department of Radiology, Imperial College Healthcare NHS Trust, London, UK; 3 Grantham Institute, Imperial College London, London, UK; 4 Department of Medical Imaging, University of Toronto, Toronto, Canada; 5 Joint Department of Medical Imaging, University Medical Imaging Toronto, University Health Network, Toronto, Canada; 6 Dipartimento di Scienze Radiologiche ed Ematologiche, Universita Cattolica del Sacro Cuore, Rome, Italy; 7 Dipartimento Diagnostica per Immagini e Radioterapia Oncologica, Policlinico Universitario A. Gemelli IRCCS, Rome, Italy; 8 Dipartimento Diagnostica per Immagini e Radioterapia Oncologica, Fondazione Policlinico Universitario A. Gemelli IRCCS, Rome, Italy; 9 Division of Surgery and Interventional Science, University College Hospital, London, UK; 10 Department of Surgery and Cancer, Faculty of Medicine, Imperial College London, London, UK

**Keywords:** Tomography (x-ray computed), Sustainability, Endometrial cancer

## Abstract

**Objectives:**

Medical image data storage and associated greenhouse gas (GHG) emissions are increasing. We aimed to measure non-essential storage and model mitigation strategies.

**Materials and methods:**

The proportion of stored post-processed series (reformats and reconstructions) was retrospectively recorded in 183 baseline staging CT chest–abdomen–pelvis studies (CT-CAP) for endometrial cancer in a UK referral centre between 2013 and 2016 (Cohort A). File size (megabytes, MB) of each series was recorded for 30 studies (Cohort B) and compared with 100 Canadian studies (Cohort C), contextualised by a survey of protocols across 17 global centres (including Cohort C). Storage-associated GHG emissions were modelled over 20 years for various mitigation strategies.

**Results:**

Post-processed series were stored in 179/183 (97%) of cohort A, 29/30 (97%) of cohort B and 16/17 (94%) of global centres. Median file size was 787 MB (IQR 460, 1257) for the entire CT study (all stored series) and 290 MB (224, 355) for the acquired axial series alone. On-premises storage of all series for new UK endometrial cancer baseline studies 2020–2040 is estimated to generate 381 metric tons CO_2_ equivalent (MTCO_2_e). Over this period, modelled mitigation strategies achieved emission reductions of 69% by storing only acquired axial series (117MTCO_2_e), 82% combining axial-only with cloud storage (70MTCO_2_e), 81% combining axial-only with an 8-year data retention policy (72MTCO_2_e), and 89% combining all three strategies (43MTCO_2_e).

**Conclusion:**

CT data storage has a large environmental cost, necessitating global action. Various mitigation strategies are achievable in reducing storage-related emissions by up to 89%.

**Key Points:**

***Question*** *Storage of non-essential post-processed CT image series contributes significantly to the accumulating image data storage-associated GHG emissions burden*.

***Findings*** *Modelling predicts emission savings of 69% by avoiding non-essential series storage in staging CTs of UK endometrial cancer patients, with comparable savings globally, based on current practice*.

***Clinical relevance*** *GHG emissions can be substantially reduced by not storing non-essential CT reformats, a mitigation that can be implemented immediately by radiologists. Further GHG mitigation is achievable using cloud storage and data-retention policies*.

**Graphical Abstract:**

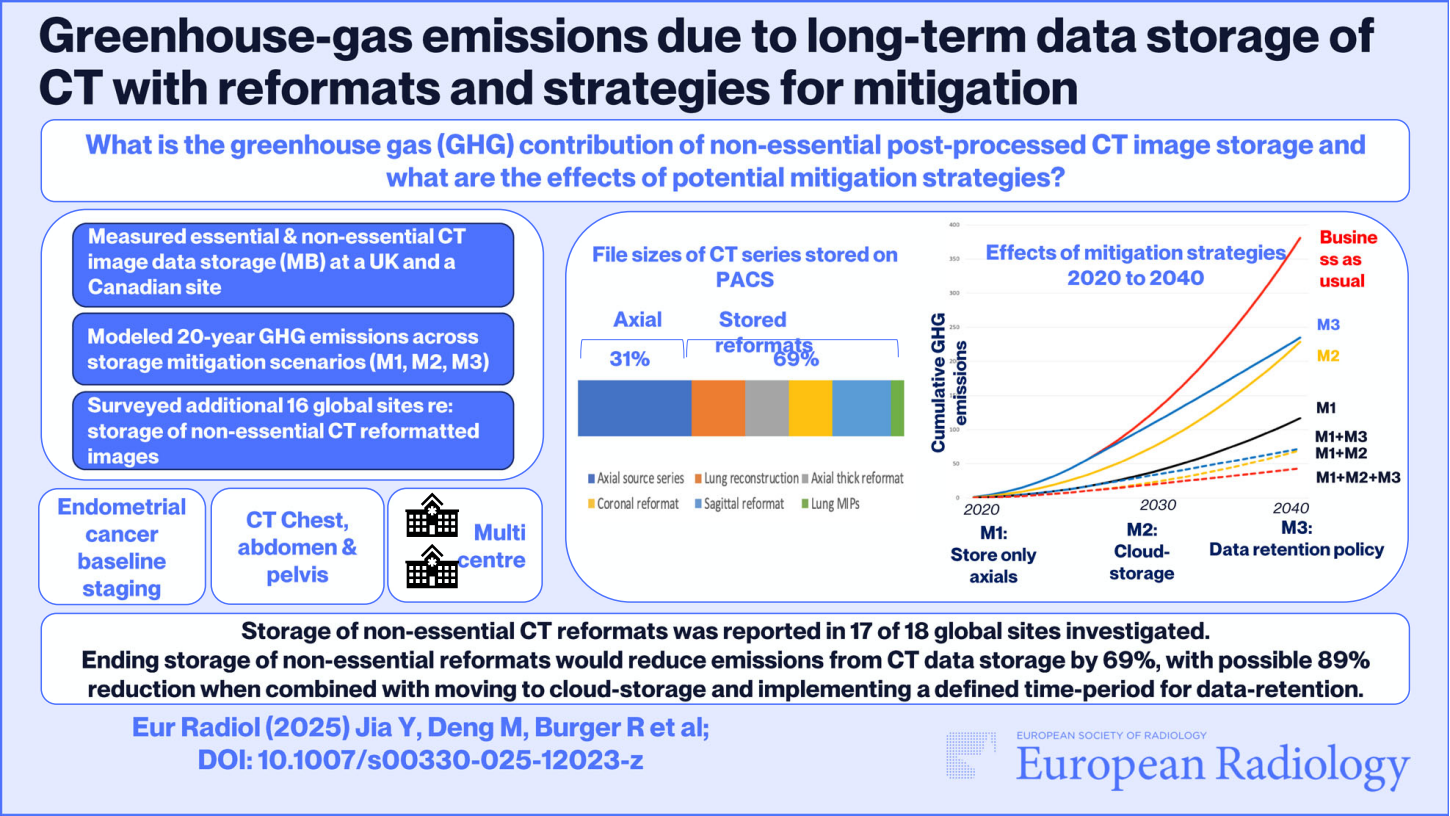

## Introduction

The 2022 United Nations Sustainable Development Goals Report highlights the critical need for immediate action to cut greenhouse gas (GHG) emissions [1]. The entire medical imaging sector is estimated to contribute up to 1% of total global GHG emissions [2, 3].

While several studies have evaluated the environmental footprint of radiology equipment, image acquisition, digital communication, and supplies, image data storage-related emissions remain under-explored [4–9]. Data storage systems generate carbon emissions through continuous energy consumption for operations and cooling, as well as during the full lifecycle of hardware from manufacturing to disposal.

Many factors drive the increasing storage burden, including the growing demand for and complexity of imaging studies, and the expanding need for labelled image datasets to support artificial intelligence applications [10–12]. For example, the Irish national radiologic data system reported over one petabyte of stored data in 2020 (23% annual growth rate over the previous five years), with CT studies growing from 66 MB (2011) to 160 MB (2020), accounting for 53% of storage in 2020 [13].

This study evaluates strategies for minimising the GHG emissions of stored imaging data, primarily in clinical settings, though similar strategies can be applied to research settings. The increasing size of CT studies partly results from the storage of multiple post-processed series, including reformats and reconstructions [13]. Historically, reformats were stored as hard-copy film due to on-demand processing being time-consuming. Modern Picture Archiving and Communication System (PACS) can now instantaneously generate quality reformats from original axial acquisitions. While certain specialised scanner-level reconstructions (e.g. high-resolution lung) contain unique diagnostic information and cannot be replicated, standard reformats/duplicates represent unnecessary storage that increases GHG emissions without clinical benefit. In addition, switching from on-premises to cloud storage, and a strategy for sustainable long-term archiving, especially beyond the life of the subject, needs careful consideration.

This study aimed to estimate the GHG emissions associated with CT reformat storage using endometrial cancer (EC) baseline staging CT measurements, modelled over 20 years [14]. Secondary aims were to explore global imaging storage protocols, compare on-premises with on-cloud storage-associated emissions, and model the GHG impact of a hypothetical data-retention policy. Projections to colorectal cancer data were included for illustration.

## Materials and methods

### Datasets

Institutional approval was obtained for this quality improvement project at the primary UK site (Audit registration number 927), using a previously anonymised dataset. We retrospectively reviewed 183 consecutive standard-of-care portal-venous CT chest, abdomen and pelvis (CT-CAP) scans performed for baseline EC staging between 2013-2016 (Cohort A), recording the site and date of acquisition, and which post-processed series (reformats, reconstructions, and duplicates) were stored for each scan. As a tertiary referral gynae-oncology centre, the CT scans reviewed were from multiple sites, with differing reformat protocols. To allow for direct comparison, scans were only included if the full CAP were acquired on the same attendance.

We recorded the file size of each acquired axial series and post-processed series for the first 30 CT scans from cohort A (Jan 2013 to Dec 2014; Cohort B). A subset was used rather than the entire Cohort A for file size analysis due to the time-intensive nature of manual file size measurement, and since there was limited file size variation, this subset was sufficient to demonstrate the scale of the problem in this exploratory analysis.

To demonstrate global applicability, an external Canadian site provided data on 100 sequential baseline EC staging CT-CAP scans from 2024 (Cohort C), including file sizes per series for 20 randomly selected scans from this cohort (Cohort D). An electronic survey was also distributed to members of the European Society of Urogenital Radiology (ESUR) female pelvic imaging group and global gynaecologic centres in January-February 2025 (Supplementary Material—Survey). Sites reported: (1) which post-processed series were stored routinely and (2) if external images are reviewed at the multidisciplinary tumour board (MDT), which series were stored for those reviewed the previous week (up to 10).

### Data analysis

The mean file size of each series type from the subset (Cohort B; *n* = 30), combined with the percentage of studies with each series type in the entire cohort (Cohort A; *n* = 183), was used for subsequent modelling. Three storage scenarios were evaluated, including only the acquired axial series, with additional lung reconstructions, or with all additional series.

### Cancer incidence projection and capacity modelling

Annual endometrial and colorectal cancer incidence was projected over 20 years (2020–2040) for UK, continental Europe, North America, Australia, and China, to allow wider modelling of GHG emissions and mitigation effects [11, 15]. Colorectal cancer was chosen as a common cancer worldwide that typically also uses baseline CT-CAP staging, where excess post-processed series storage is non-essential. Linear regression was applied to normalise geographical variations and cancer case projections.

A custom capacity model was developed in Microsoft Excel to estimate the storage demand for the three storage scenarios. Total raw data capacity, measured in Terabytes, projected over 2020–2040, was calculated based on the initial data size (Cohort A and B) and the multi-region projection of annual cancer incidence. Security backups, which are typically stored across multiple distinct locations and protocols vary significantly between centres, are not included in this model.

### Modelling storage-associated electricity consumption and resulting GHG emissions using on-premises storage

#### Energy model

The energy consumption model used a hardware-centric approach to calculate baseline IT energy consumption (*P*_IT_) from three components: storage systems (the physical data storage devices; hard drives at 136 kWh/TB/year), servers (used to process data and run applications; 12TB capacity/server, 2059 kWh/server/year), and network infrastructure (hardware required for servers to connect to each other and the internet; 0.1device/server, 3197 kWh/device/year) [16–19]. Total facility energy consumption (*P*_total_) was calculated by multiplying *P*_IT_ by a power usage effectiveness (PUE) to account for infrastructure energy consumption (supporting systems, e.g. transformers, Uninterruptible Power Supplies, lighting, and cooling) [18]. A PUE of 1.8 was used, consistent with average European data centres [20]. Additional energy consumption due to transmission and distribution losses (8%, 1.7% of *P*_total_, respectively) was included [21].

### Environmental impact assessment and projection

Emissions were calculated from the energy consumption using the 2024 emission conversion factors for each region (Supplementary Table 1) [22, 23].

Scope 1 (direct emissions—e.g. fuel combustion, refrigerant leakage) and Scope 3 GHG emissions other than transmission and distribution losses (value chain emissions—e.g. production, maintenance, and disposal of hardware and buildings, extraction and processing of fuels) were not included in this model. Scope 1 emissions are largely negligible (< 1%) for data centres; modelling Scope 3 emissions requires extensive supplier data that is typically unavailable, varies significantly between centres, and extends beyond the boundaries of this paper.

GHG emissions were evaluated as metric tons of carbon dioxide equivalent (MTCO_2_e). Carbon dioxide equivalent (CO_2_e) represents an amount of a GHG whose atmospheric impact is standardised to one unit of mass of carbon dioxide (CO_2_), based on the global warming potential of the gas. This is consistent with scientific consensus for reporting and allows for comparison with other sources of healthcare-related emissions.

This model assumes that file sizes and prevalence of stored series, data centre electricity consumption, and GHG emission conversion factors remain unchanged.

### Impact of other GHG mitigation strategies

Assumptions for GHG mitigation by switching to cloud were informed by available data (Supplementary Table 2). Exact figures vary between published sources. An independent report calculated carbon intensity (in MTCO_2_E/workload) from stand-alone US data centres and hyperscale cloud providers to be 0.75 and 0.15, respectively, an 80% reduction, which we used as the higher estimate in our model [24]. The lower 40% estimate used in the model is derived from a 2010 Microsoft report that identified a decrease of 30–60% in emissions by comparing large-scale on-premises deployment to equivalent cloud deployment.

For illustration, we additionally modelled a short data-retention policy of 8 years followed by transfer to a place of deposit [25]. Based on this, GHG emissions for each storage scenario were calculated assuming all files were transferred to deep storage with negligible energy consumption eight years after creation [24, 26].

All descriptive statistics were summarised as median (25th percentile, 75th percentile), mean ± standard deviation, and range.

## Results

### Overview of the dataset

In Cohort A, 66% of studies (120/183) were acquired at the UK referral site, with the remaining cases sent from 17 other UK centres. 97% (179/183) of studies stored at least one post-processed series. Coronal reformats (91%), sagittal reformats (86%), and lung reconstructions (85%) were most common, with similar proportions in cohort B (87%, 70%, and 70%). There were further duplicated reformats or reconstructions (cohort A *n* = 6), and additional non-contrast (cohort A *n* = 3, and cohort B *n* = 1) and arterial phase series (cohort A *n* = 9 and cohort B *n* = 2).

**Fig. 1:**
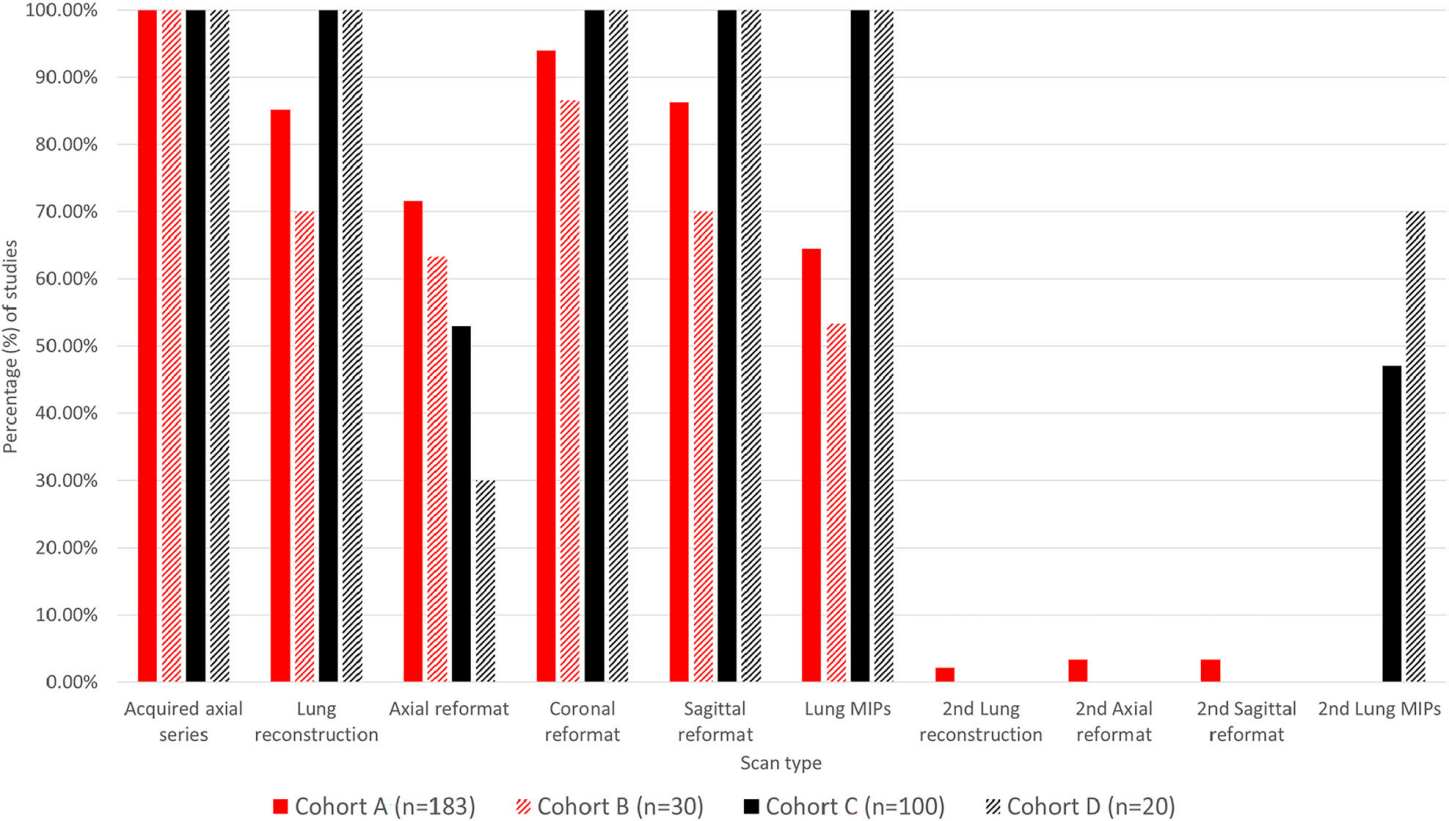
Percentage of studies with each portal-venous phase scan type stored on PACS. Cohort A (*n* = 183) includes all available staging studies for endometrial cancer patients at the primary UK site between 2013 and 2016. Cohort B (*n* = 30) is a subset of cohort A, representing the first 30 of these studies, in which a detailed analysis of file sizes was undertaken. Cohort C (*n* = 100) includes 100 sequential staging studies for endometrial cancer patients in an external Canadian site in 2024. Axial reformats refer to both thick axial series and duplicate copies of the thin axial series. Cohort D (*n* = 20) is a subset of cohort C, representing 20 randomly selected studies from cohort C, in which a detailed analysis of file sizes was undertaken. Axial reformats refer to both thick axial series and duplicate copies of the thin axial series. MIPs, maximum intensity projections

In Cohort C, 100% of studies had stored post-processed series (Fig. 1). Survey responses were obtained from 16 centres (10 countries): Italy (4), China (2), the UK (2), the USA (2), and one each from France, Japan, Portugal, Spain, Sweden, and Switzerland. Including Cohort C, 16/17 (94%) international centres stored post-processed series. External CTs sent to referral centres for MDT all had at least one stored post-processed series (Supplementary Table 3).

### File size analysis

In Cohort B (*n* = 30), the non-contrast (*n* = 1) and arterial phase (*n* = 2) sequences accounted for 6% of the total size of the cohort. These files were excluded from further modelling due to the small numbers.

**Fig. 2:**
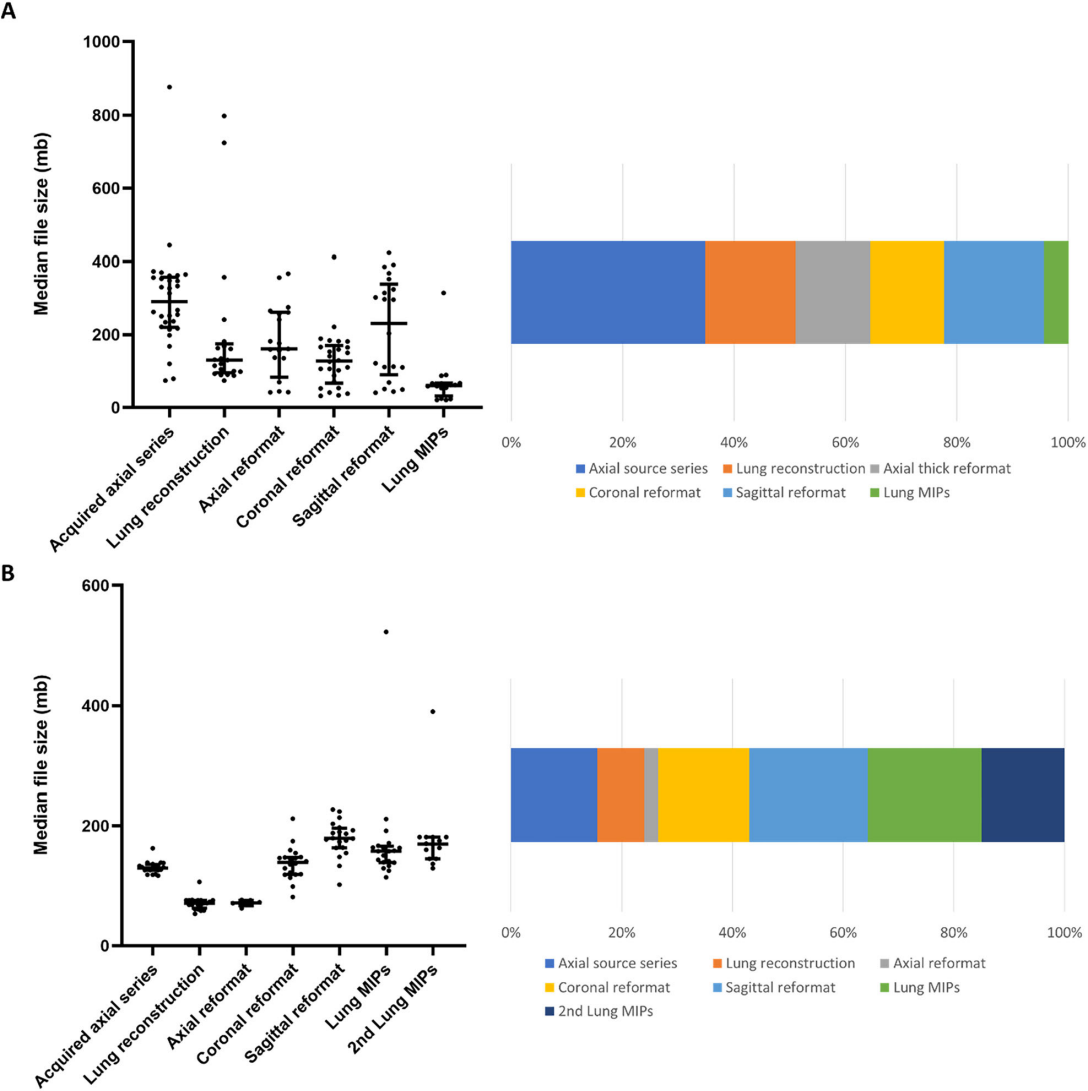
File size of each series type stored on PACS in Cohort B (*n* = 30) and Cohort D (*n* = 20). **A** Cohort B (*n* = 30) represents a subset containing the first 30 of all available complete portal-venous phase staging CT-CAP studies for endometrial cancer patients in the primary UK site between 2013 and 2016. **B** Cohort D (*n* = 20) is a subset containing a randomly selected 20 complete portal-venous phase staging CT-CAP studies for endometrial cancer patients in the Canadian centre in 2024. Left: median file sizes (megabytes) for each type with interquartile range bars. Right: the total size of each scan type as a percentage of the total stored size for all portal-venous phase CT files. Axial reformats refer to both thick axial series and duplicate copies of the thin axial series. PACS, picture archiving and communication system; MIPs, maximum intensity projections; MB, megabyte; CT-CAP, CT chest, abdomen and pelvis

For portal-venous phase acquisition, the median file size of the entire CT study was 787 (25th, 75th percentiles = 460, 1257) MB. The median file size of the acquired axial thin-slice series was 290 (224, 355) MB. Of the stored post-processed series, the sagittal reformat had the largest median file size of 231 (110, 324) MB, followed by duplicate axial reformats with a median size of 161 (109, 259) MB. The sagittal and duplicate axial reformats represented 18% and 13% of the total file size, respectively (Table 1 and Fig. 2A).

**Table 1:** File sizes stored on PACS in cohort B (UK centre; *n* = 30), cohort D (Canadian centre; *n* = 20) and projected file sizes for Cohort A (*n* = 183) for each series type

	Portal-venous phase total (MB)	Acquired axial series	Lung reconstruction	Axial reformats	Coronal reformat	Sagittal reformat	Lung MIPs	2nd Lung MIPs
Cohort B (*n* = 30)	25,590 (100%)	8906 (35%)	4161 (16%)	3409 (13%)	3392 (13%)	4595 (18%)	1128 (4%)	NA
Mean ± SD file size (MB)	853 ± 447	297 ± 142	198 ± 198	179 ± 100	130 ± 80	219 ± 145	70 ± 68	NA
Median (IQR) file size (MB)	787 (460, 1257)	290 (224, 355)	130 (99, 168)	161 (109, 259)	128 (75, 166)	231 (110, 324)	60 (46, 66)	NA
Slice thickness in mm (mean/median (range))	Not applicable	1.7/1.5 (0.625–3)	1.5/1.5 (1.0–2.0)	5.1/5.0 (2.5–8)	2.6/2.0 (1.5–5.0)	2.5/2.0 (1.5–5.0)	7.8/8.0 (5.0–8.0)	NA
Cohort A (*n* = 183)	177,245 (100%)	54,327 (31%)	30,907 (17%)	23,503 (13%)	22,437 (13%)	34,572 (20%)	8316 (5%)	NA
Cohort D (*n* = 20)	16,792 (100%)	2611 (16%)	1416 (8%)	426 (3%)	2751 (16%)	3574 (21%)	3445 (21%)	2512 (15%)
Mean ± SD file size (MB)	840 ± 194	131 ± 10	71 ± 11	71 ± 5	138 ± 28	179 ± 63	172 ± 86	179 ± 63
Median (IQR) file size (MB)	790 (759, 862)	130 (126, 134)	71 (66, 76)	72 (69, 75)	139 (119, 147)	179 (164, 194)	158 (139, 165)	170 (149, 181)
Slice thickness in mm	Not applicable	3 or 5*	3	3*	3	3	8	8
Cohort C (*n* = 100)	81,189 (100%)	13,057 (16%)	7081 (9%)	3767 (5%)	13,753 (17%)	17,872 (22%)	17,227 (21%)	8432 (10%)

Cohort B (*n* = 30) is a subset containing the first 30 staging studies (by date) for endometrial cancer patients from cohort A. Additional stored non-contrast (*n* = 1) and arterial phase (*n* = 2) series, which accounted for 6% (1737/27,327 MB) of the total stored data, were excluded due to the small number of these stored series. File sizes were estimated for Cohort A based on the mean file sizes of Cohort B and the percentage of studies with each scan type in Cohort A

Cohort D (*n* = 20) is a subset containing a random 20 staging studies for endometrial cancer patients from cohort C (Canadian centre 2024; *n* = 100). One study additionally stored single-energy metal artefact reduction (SEMAR) axial series, which accounted for 0.3% (56/16792 MB) of total stored data. File sizes were estimated for Cohort C based on the mean file sizes of Cohort D and the percentage of studies with each scan type in Cohort C.

The top row displays the sum of all studies or series types in this cohort, with their percentage contribution in relation to all stored data. Axial reformats refer to both thick axial series and duplicate copies of the thin axial series. Descriptive statistics per study and per series type are displayed as mean ± standard deviation (SD) and median (25th percentile, 75th percentile). All values for file sizes are displayed to zero decimal points.

* There were two distinct storage protocols for axial series in Cohort D: (1) chest, abdomen, pelvis all stored as 3 mm slices (14/20) and (2) chest, abdomen, pelvis all stored as 5 mm slices, with chest also stored as 3 mm slices (6/20)

*MIPs* maximum intensity projections, *mm* millimetre, *MB* megabyte

Cohort D (Canadian centre; *n* = 20) had a similar median total file size of 790 (759, 862) MB. The acquired axial series had a smaller median file size of 130 (126, 134) MB and occupied only 16% of the total file size (Table 1 and Fig. 2B). Notably, Cohort D had a greater slice thickness of stored axial series in Cohort D (3 mm or 5 mm for all studies, compared to median thickness of 1.5 mm in Cohort B).

### Modelling of potential savings in GHG emissions from updating reformat storage protocols

There were 11,385 new endometrial cancer patients in the UK in 2020 [15]. According to the analysed file sizes and reformat proportions, storing staging CT-CAP studies for these requires 11,027 GB [14]. If all studies were stored indefinitely, using on-premises storage, these would cost 7.3 MWh/year, producing 1.5 MTCO_2_e/year (Supplementary Fig. 1). Over 20 years (2020–2040), new endometrial cancer cases in the UK are estimated to be 265,000, cumulatively requiring 257,000 Gb of storage, costing 181 MWh, and producing 381 MTCO_2_e of storage-associated emissions (Table 2).

**Table 2:** Cumulative GHG emission associated with the storage of new endometrial cancer staging CT-CAP studies projected from 2020 to 2040, with mitigation by reducing reformat storage and migration to cloud storage

Region	Number of new cases		Acquired axial series + all reformats and duplicates					Acquired axial series + Lung reconstructions						Acquired axial series only					
	2020	Projected cumulative 2020–2040	On-premises storage (MTCO_2_e)	Cloud storage (MTCO_2_e (%decrease))				On-premises storage (MTCO_2_e (%decrease))		Cloud storage (MTCO_2_e (%decrease))				On-premises storage (MTCO_2_e (%decrease))		Cloud storage (MTCO_2_e (%decrease))			
UK	11,385	265,203	381	229	(40%)	76	(80%)	183	(52%)	110	(71%)	37	(90%)	117	(69%)	70	(82%)	23	(94%)
AU	3055	76,430	281	169	(40%)	56	(80%)	135	(52%)	81	(71%)	27	(90%)	86	(69%)	52	(82%)	17	(94%)
CN	81,964	1,888,388	7275	4365	(40%)	1455	(80%)	3499	(52%)	2099	(71%)	700	(90%)	2230	(69%)	1338	(82%)	446	(94%)
EU	188,583	2,634,564	5186	3112	(40%)	1037	(80%)	2494	(52%)	1496	(71%)	499	(90%)	1590	(69%)	954	(82%)	318	(94%)
NA	68,378	1,579,239	3912	2347	(40%)	782	(80%)	1881	(52%)	1129	(71%)	376	(90%)	1199	(69%)	719	(82%)	240	(94%)
Total	271,980	617,8621	16,654	9992	(40%)	3331	(80%)	8009	(52%)	4805	(71%)	1602	(90%)	5105	(69%)	3063	(82%)	1021	(94%)

Projected figures assume all files are stored indefinitely. Projected figures for cloud storage assume a 40% reduction in GHG as a conservative estimate, or an 80% reduction in line with recent reports (see Supplementary Table 2). The % decrease represents GHG savings relative to if acquired axial series + all reformats and duplicates were stored indefinitely on-premise. Emissions were calculated based on the mean file sizes of Cohort B (*n* = 30; a subset of Cohort A), the percentage of studies with each scan type in Cohort A (*n* = 183), associated total energy consumption (internal storage, server, network, and infrastructure), and the emission conversion factor for each region [22]. Cancer incidence statistics were derived from GLOBALCAN [11, 15]. All numbers are displayed to zero decimal points

*MTCO2e* metric tons CO_2_ equivalent, *CTCAP* CT chest abdomen and pelvis, *UK* United Kingdom, *AU* Australia, *CN* China, *EU* European Union, *NA* North America

**Fig. 3:**
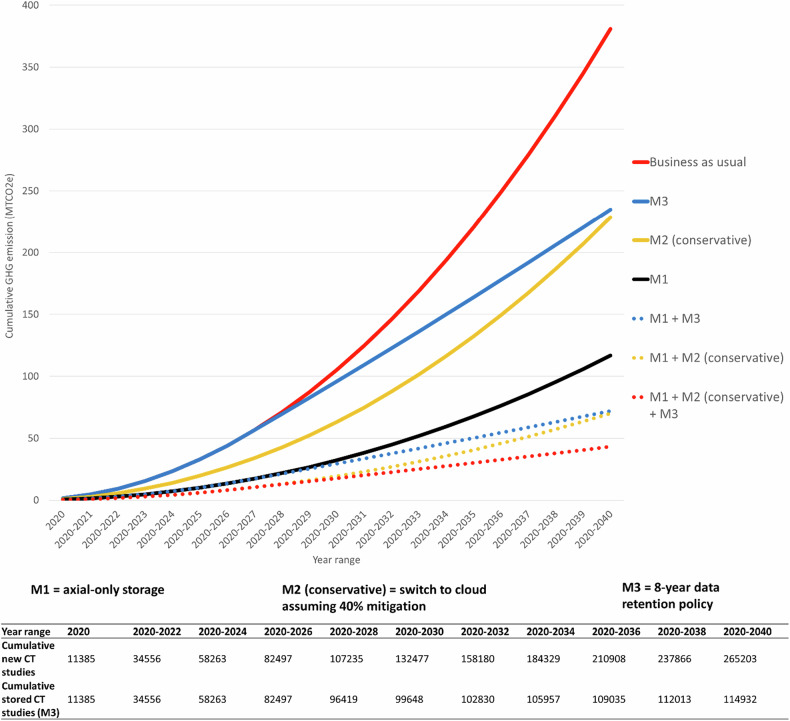
GHG emissions associated with the storage of new endometrial cancer staging CT-CAP studies projected from 2020 to 2040 in the UK with various mitigation strategies. Displayed as cumulative carbon emission. Business as usual assumes all files are stored according to current file sizes and proportions, on-premises, and indefinitely. Mitigation 1 (M1): storage of only the acquired axial series. M2 (conservative): switch to cloud storage based on conservative mitigation of 40%. M3: simplified data retention policy of moving to a permanent archive (with negligible electricity consumption) 8 years after acquisition. The number of cumulative new studies and cumulative stored studies (if M3 was implemented) over this period is displayed in the bottom table. Emissions were calculated based on the mean file sizes of Cohort B (*n* = 30; a subset of Cohort A), the percentage of studies with each scan type in Cohort A (*n* = 183), and associated total energy consumption (internal storage, server, network, and infrastructure). Cancer incidence statistics were derived from GLOBALCAN [7, 10]. MTCO_2_e, metric tons CO_2_ equivalent; CT-CAP, CT chest abdomen and pelvis; UK, United Kingdom

Avoiding storage of all post-processed series would mean that the staging CT-CAP studies for new cases in 2020–2040 would require only 78,700 GB of storage, saving 264MTCO_2_e over 20 years (69% decrease). If lung reconstructions were also stored, new cases in 2020–2040 would require 124,000 GB of storage, saving 198 MTCO_2_e over 20 years (52% decrease) (Fig. 3 and Table 2). If all evaluated regions avoided storage of all reformats during this period, 11,549 MTCO_2_e could be saved (or 8645 MTCO_2_e if lung reconstructions were also stored).

Given the higher incidence of colorectal cancer across all regions, and similar baseline staging CT-CAP requirements, the potential GHG emissions avoided are even greater for this indication. In the UK alone, storing only the acquired axial series for colorectal cancer staging CTs would save 813MTCO_2_e between 2020–2040 (or 609 MTCO_2_e if lung reconstructions were also kept). Across multiple global regions, this would save 36,687 MTCO_2_e (or 27,462 MTCO_2_e if lung reconstructions were also kept) (Supplementary Fig. 3 and Supplementary Table 4).

### Modelling other GHG mitigation strategies

Migrating all studies to cloud storage would reduce GHG emissions by 40–80% annually. Combining this with avoiding reformat storage would reduce the storage-associated emission for new endometrial cancer patients in the UK during this period by 82–94% (a reduction of 311–358 MTCO_2_e) or 71–90% (a reduction of 271-344 MTCO_2_e) if lung reconstructions were kept in addition (Table 2).

If all post-processed series were stored, but a short data-retention policy of 8 years followed by transfer to a place of deposit was applied, the storage of staging CT-CAP studies for new endometrial cancer patients in the UK would produce 235 MTCO_2_e between 2020 and 2040, a reduction of 146 MTCO_2_e (38%). Combining this with avoiding reformat storage would reduce the storage-associated GHG emissions for new endometrial cancer patients in the UK during this period by 81% (309 MTCO_2_e) or 70% (268 MTCO_2_e) if lung reconstructions were kept in addition (Fig. 3 and Table 3).

**Table 3:** Cumulative GHG emission associated with the storage of new endometrial cancer staging CTCAP studies projected from 2020 to 2040, with mitigation by reducing reformat storage and a simplified data retention strategy

Region	Number of new cases		Acquired axial series + all reformats and duplicates			Acquired axial series + Lung reconstructions				Acquired axial series only			
	2020	Projected cumulative 2020–2040	No image retention policy (MTCO_2_e)	Deep storage after eight years (MTCO_2_e (%decrease))		No image retention policy (MTCO_2_e (%decrease))		Deep storage after eight years (MTCO_2_e (%decrease))		No image retention policy (MTCO_2_e (%decrease))		Deep storage after eight years (MTCO_2_e (%decrease))	
UK	11,385	265,203	381	235	(38%)	183	(52%)	113	(70%)	117	(69%)	72	(81%)
AU	3055	76,430	281	175	(38%)	135	(52%)	84	(70%)	86	(69%)	54	(81%)
CN	81,964	1,888,388	7275	4469	(39%)	3499	(52%)	2149	(70%)	2230	(69%)	1370	(81%)
EU	188,583	2,634,564	5186	3171	(39%)	2494	(52%)	1525	(71%)	1590	(69%)	972	(81%)
NA	68,378	1,579,239	3912	2407	(38%)	1881	(52%)	1157	(70%)	1199	(69%)	738	(81%)
Total	271,980	6,178,621	16,654	10,222	(39%)	8009	(52%)	4915	(70%)	5105	(69%)	3134	(81%)

All figures assume storage on-premises. Projected figures for the no image retention policy assume all files are stored indefinitely. Projected figures for Deep storage after eight years assume negligible electricity consumption in deep storage using hard drives. The % decrease represents carbon savings relative to if acquired axial series + all reformats and duplicates were stored indefinitely. Emissions were calculated based on the mean file sizes of Cohort B (*n* = 30; a subset of Cohort A), the percentage of studies with each scan type in Cohort A (*n* = 183), associated total energy consumption (internal storage, server, network, and infrastructure), and the emission conversion factor for each region [22]. Cancer incidence statistics were derived from GLOBALCAN [11, 15]. All numbers are displayed to zero decimal points

*MTCO2e* metric tons CO_2_ equivalent, *CTCAP* CT chest, abdomen and pelvis, *UK* United Kingdom, *AU* Australia, *CN* China, *EU* European Union, *NA* North America

Combining all three mitigation strategies (axial-only storage, moving to cloud storage, and an 8-year data-retention policy) would reduce the storage-associated GHG emissions for new endometrial cancer patients in the UK during this period by 89% (338 MTCO_2_e) (Supplementary Table 6).

The GHG mitigation from implementing these strategies for colorectal cancer is even greater due to its higher incidence (Supplementary Tables 4, 5, and 7).

## Discussion

This study aimed to measure non-essential data storage and model mitigation strategies for rapidly growing storage-related GHG emissions.

Storage of multiple reformatted and reconstructed images for baseline staging CTs in endometrial cancer was a routine practice globally in nearly all centres surveyed in 2025. Our model projected GHG emissions of 381 MTCO_2_e for the storage of baseline CT in new endometrial cancer in the UK from 2020 to 2040, with a reduction of nearly 70% (264 MTCO_2_e), to 117 MTCO_2_e, by only storing the acquired axial series. Reductions are far more substantial when extrapolated globally due to greater disease burden and/or higher emission conversion factors. Transfer of data storage to the cloud would reduce emissions by 40% (conservative estimate) to 80% (optimistic estimate). Implementing a data-retention policy of deep storage after 8 years from the date of CT acquisition, estimated savings of 38% if all series were stored, and 81% if combined with axial-only storage.

Only storing the acquired axial images in UK new endometrial and colorectal cancer cases between 2020–2040 would save 1077 MTCO_2_e, equivalent to the cumulative electricity consumption of 102 homes over 20 years [27] (Supplementary Methods). These savings are in line with other energy-saving mitigations, such as switching off computers [6, 28]. Improving the environmental impact of radiology requires mitigation in all operational areas, including reducing inappropriate imaging through decision support tools, use-phase emissions through protocol efficiency and imaging acceleration, and non-productive energy waste through automated low-power modes [29–31]. Image storage strategies can be applied via automated workflow, requiring no ongoing individual effort, unlike other mitigations, which require organisational and behavioural change. Applying these mitigation strategies to other imaging modalities and clinical scenarios will have cumulative benefits.

Higher post-processed series storage proportions in Cohort C and the global survey suggest even greater potential GHG savings from mitigation strategies. Cohort D’s significantly smaller acquired axial file sizes (130 MB vs 290 MB in Cohort B) resulted from thicker slice acquisition (3-5 mm vs 1.5 mm; Table 1). However, this thicker slice acquisition may limit reformat quality and explain the compensatory storage of additional post-processed series. Institutions should evaluate optimal axial slice thickness for high-quality reformats, which likely differs by modality and indication.

Given the compounding imaging burden, the need for large, robust, and secure data storage is pressing [32]. Cloud-based solutions may form the basis for the next generation of radiology infrastructure as they are generally more energy efficient than on-premise options [24], though their benefits and disadvantages need to be considered for individual sites [33, 34]. Publicly owned “trusted research environments” (TRE) are an emerging option for cloud-based storage, potentially matching the power efficiency of commercial platforms whilst resolving issues concerning data privacy, security, and ownership associated with entrusting medical data to the latter [35].

Comprehensive image retention guidelines from relevant regulatory bodies are a complementary strategy in minimising storage-associated emissions. Guidelines must consider the needs of different patient groups, such as paediatrics or oncology, patient vital status, as per the NHS guidelines [25], as well as ethical, clinical, and economic factors, and future research needs. Longer-term planning should consider appropriate low-energy archiving of ‘dark data’ as an intermediate step towards eventual data deletion after a defined period. Without deletion, total storage requirements will continue to grow indefinitely, incurring significant Scope 3 GHG emissions.

Our study’s limitations include the use of a selected patient dataset from a single UK centre (though studies were sent from 17 sites) for model development. File sizes and reformats/duplicates proportions differ across regions, clinical indications, and modalities. This was evident in our Canadian cohort and the present-day global survey. The GHG associated with generating reformats compared with retrieving from storage at the point of use was outside the boundary of this study, but could be considered. We found limited research data on the mitigation of emissions from switching to cloud, limiting the strength of the assumptions; thus, we provided both conservative and optimistic predictions.

Similarly, storage-associated electricity consumption and GHG emissions vary between geographic regions, depending on differences in electricity efficiency and local carbon intensity. We did not address the impact of radiology departments transitioning to carbon-neutral energy sources, nor the storage requirements and GHG emissions associated with datasets for AI model training and image processing [36]. Emissions associated with storing multiple security backups of imaging studies were not included due to significant protocol variations, so applying the mitigations to these backups would yield further significant savings. Scope 3 emissions account for a significant portion of a data centre’s carbon footprint, though they were not included in the model, suggesting even greater environmental benefits if lifecycle emissions were fully accounted for [37].

Despite these limitations, the implications are clear. Given the exponential accumulation of medical imaging data, there is an urgent need to streamline storage protocols. This study provides data on the projected GHG emissions related to the storage of non-essential reformats. Avoiding non-essential data storage is an easily implementable and scalable, with similar or greater magnitude compared to other mitigation strategies in radiology. Global collaborative action is needed to improve sustainability in radiology, and improved data storage represents an important strategy in achieving this goal [38, 39].

## Supplementary information

Supplementary information

## Data Availability

No individual deidentified participant data will be shared. We currently do not have institutional permission to share the model or data, but are working to secure this.

## Abbreviations

CT-CAP: CT of chest, abdomen and pelvis
GHG: Greenhouse gas
MTCO_2_e: Metric tons CO_2_ equivalent
