# Supplementary material for: Greenhouse gas emissions due to long-term data storage of CT with reformats and strategies for mitigation
Source: Eur Radiol. 2025 Sep 30;36(3):2186–97. doi: 10.1007/s00330-025-12023-z (PMC12963087; doi:10.1007/s00330-025-12023-z)
Supplement: Supplementary file 1 — Supplementary information [file 330_2025_12023_MOESM1_ESM.pdf]

Greenhouse-gas emissions due to long-term data storage of CT with reformats and strategies for mitigation

ELECTRONIC SUPPLEMENTARY MATERIAL

Eur Radiol (2025) Jia Y, Deng M, Burger R, et al.

Supplementary methods – modelling electricity consumption of homes in the UK

The median electricity consumption for all households in the UK was 3100 kWh/year in 2018 and falling by 1.875% per year (1). Based on this, the average electricity consumption per household per year from 2020-2040 would be 2491.5 (since the 3100 figure is from 2018, the 2020 electricity consumption would be 2985.9). Using the Department for Environment, Food & Rural Affairs (DEFRA) emission conversion factors of 0.211 kgCO_2e/kWh (2), this would be equivalent to 525.4kgCO_2e/home/year. This estimate assumes that the emission conversion factor remains constant for this duration.

	**Emission conversion factor (kgCO2e/ kWh)**
**United Kingdom**	0.211
**Australia**	0.552
**China**	0.560
**Europe**	0.284
**North America (weighted average)**	0.362
United States of America	0.384
Canada	0.175

Supplementary Table 1. Emission conversion factor per region (3 s.f.). Emissions were calculated using the 2024 emission conversion factors for each region (2). For North America (NA), cancer incidence statistics was available for United States of America and Canada only, so the emission conversion factor for NA was calculated by taking a weighted average across the two countries based on 2024 population (3).

	Reported GHG reduction	Source
US hyperscale data centres (2021)	80%	Siddik MAB, Shehabi A, Marston L (2021) The environmental footprint of data centers in the United States. Environ Res Lett 16:064017.<br><br>https://doi.org/10.1088/1748-9326/abfba1
Amazon Web Services (2024)	79%	Graham S, Yashkova O (2024) Energy and Carbon Efficiency Benefits of Public Cloud Computing over Enterprise Datacenters<br><br>https://d2908q01vomqb2.cloudfront.net/b7103ca278a75cad8f7d065acda0c2e80da0b7dc/2024/04/19/FINAL_EUR251921924_AWS_Extended_InfoBrief_V11.pdf<br><br>https://d39w7f4ix9f5s9.cloudfront.net/b0/3e/b0fc6b8a4a85b38ac65a3fbc584c/11061-aws-451research-advisory-bw-cloudefficiency-eu-2021-r5-final-corrected-data.pdf
Microsoft Azure (2020)	71-83%	Microsoft Corporation (2020) The carbon benefits of cloud computing: A study on the Microsoft Cloud in partnership with WSP<br><br>https://download.microsoft.com/download/7/3/9/739bc4ad-a855-436e-961d-9c95eb51daf9/microsoft_cloud_carbon_study_2018.pdf
Alibaba (2024)	85.5%	Alibaba Group Holding Limited (2024) Environmental, Social and Governance Report 2024. Hangzhou<br><br>https://www.alibabagroup.com/en-US/document-1752073403914780672
Microsoft Accenture Report (2010)*		Microsoft Accenture Report (2010) **Cloud Computing and Sustainability:** The Environmental Benefits of Moving to the Cloud<br><br>https://download.microsoft.com/download/a/f/f/affeb671-fa27-45cf-9373-0655247751cf/cloud%20computing%20and%20sustainability%20-%20whitepaper%20-%20nov%202010.pdf
Small on-premise deployment to cloud	>90%	
Medium on-premise deployment to cloud	60-90%	
Large on-premise deployment to cloud	30-60%	

Supplementary table 2. Reported GHG mitigation switching from on-premise data centres to cloud (hyperscale) data centres and the sources. **(**Note: No data could be found related to cloud storage using Google, Oracle or IBM).

*This report refers to savings related from transitioning computing processes (rather than storage) to cloud.

Institution	Country of practice	Hospital of practice	N of external cases	Acquired axial series	Lung reconstruction	Sagittal reformat	Coronal reformat	Axial reformat	Lung MIPs	Non-contrast	Arterial	Delayed
	Canada	Academic hospital		Yes	Yes	Yes	Yes	Yes	Yes	No	No	No
1	Spain	Academic hospital		Yes	Yes	Yes	Yes	No	No	No	No	No
			N=10	10	10	5	5	2	0	0	0	2
2	France	Academic hospital		Yes	No	No	No	No	No	No	No	No
			N=10	10	0	0	0	1	3	2	7	1
3	USA	Academic hospital		Yes	Yes	Yes	Yes	No	Yes	No	No	No
			N=10	10	10	10	10	10	10	3	0	2
4	USA	Academic hospital		Yes	Yes	Yes	Yes	Yes	No	No	No	No
			N=3	3	3	2	2	1	0	0	0	0
5	UK	Academic hospital		Yes	Yes	Yes	No	No	No	No	No	No
			N=9	9	9	6	6	9	1	0	0	0
6	Sweden	Academic hospital		Yes	No	Yes	Yes	Yes	No	No	No	No
			N=10	10	10	10	10	10	0	0	0	0
7	Japan	Academic hospital		Yes	Yes	No	Yes	Yes	No	No	No	Yes
			N=3	3	1	0	0	2	0	2	0	0
8	Switzerland	Academic hospital		Yes	Yes	Yes	Yes	No	Yes	No	Yes	No
			N=10	10	10	10	10	0	10	2	4	3
9	Italy	Academic hospital		Yes	No	Yes	Yes	Yes	No	Yes	No	No
			N=5	5	0	5	5	5	0	5	3	3
10	Portugal	Academic hospital		Yes	Yes	Yes	Yes	Yes	Yes	No	No	No
			N=10	10	10	10	10	10	10	0	0	0
11	Italy	Academic hospital		Yes	Yes	Yes	Yes	Yes	No	No	No	No
			N=2	2	2	1	1	2	1	1	0	1
12	UK	Academic hospital		Yes	Yes	No	No	No	No	No	No	No
			N=4	4	4	1	0	0	0	0	0	0
13	China	Academic hospital		Yes	Yes	Yes	Yes	Yes	Yes	Yes	Yes	Yes
			N=10	10	10	6	0	10	0	6	5	3
14	China	Academic hospital		Yes	Yes	Yes	No	Yes	No	Yes	Yes	Yes
			N=10	10	8	7	2	10	0	7	8	8
15	Italy	Academic hospital		Yes	Yes	No	No	No	No	No	Yes	No
			N=6	6	6	6	6	4	0	5	5	4
16	Italy	Academic hospital		Yes	Yes	Yes	Yes	No	Yes	Yes	Yes	Yes
			N=3	3	3	3	3	0	3	3	3	3
	Total	Protocol at centres N=17		17/17 (100%)	14/17 (82.4%)	13/17 (76.5%)	12/17 (70.6%)	9/17 (52.9%)	6/17 (35.3%)	4/17 (23.5%)	5/17 (29.4%)	4/17 (23.5%)

	Total	External scans (received by N=16 referral centres)	N=115	115/115 (100%)	98/115 (85.2%)	82/115 (71.3%)	70/115 (60.9%)	74/115 (64.3%)	38/115 (33%)	36/115 (31.3%)	35/115 (30.4%)	30/115 (26%)

Supplementary Table 3. Results of the global survey on frequency of post-processed series storage in clinical practice 2025. For each gynaecologic oncology centre, the first row describes whether the series are routinely stored, while the second row reports the number of each series available from external exams sent for review at the specialist multidisciplinary tumour board in one week, up to a maximum of 10 scans. Only one academic hospital did not include any reformatted series. All cases from external referral sites had at least one reformatted series.

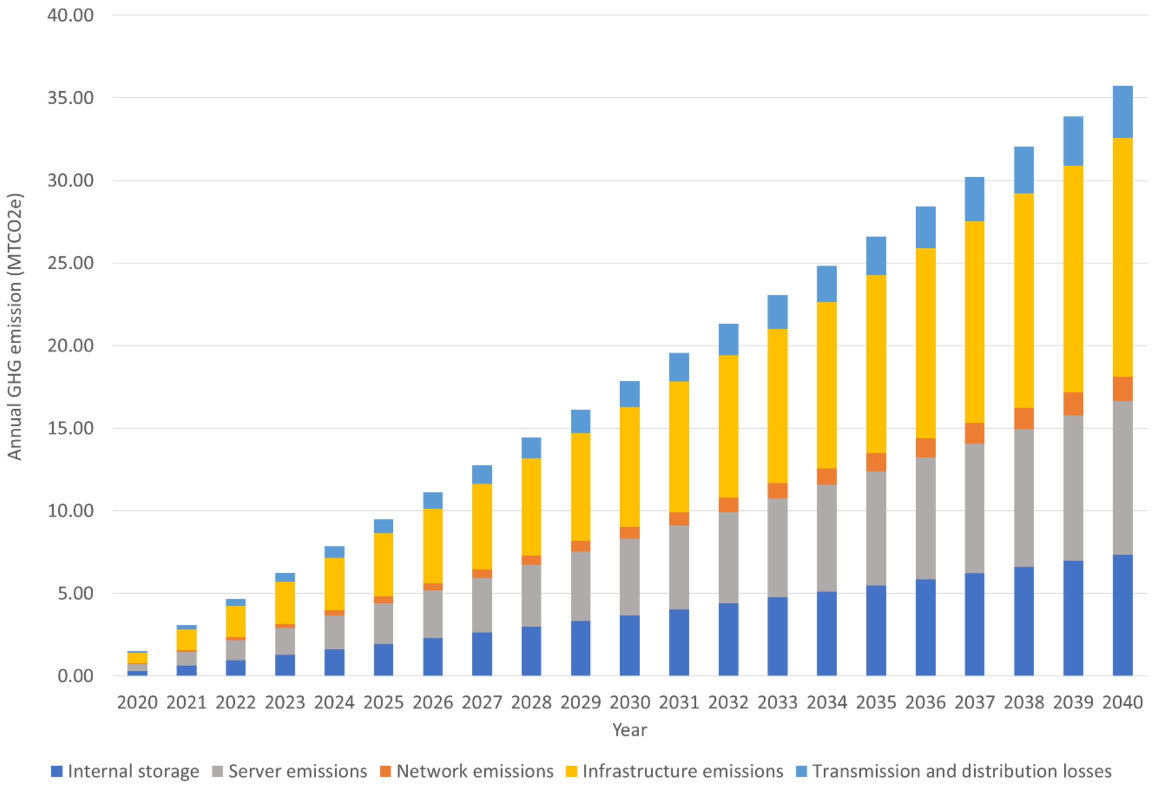

Year range	2020	20-22	20-24	20-26	20-28	20-30	20-32	20-34	20-36	20-38	20-40
Cumulative new and stored CT studies	11385	34556	58263	82497	107235	132477	158180	184329	210908	237866	265203
Cumulative storage required	11027	33470	56431	79903	103863	128311	153205	178532	204275	230386	256863

Supplementary Figure 1. Relative contribution of each source to annual greenhouse gas emissions in an on-premise data centre, projected over 20 years.

Projected emissions reflect those associated with the storage of new endometrial cancer staging CT-CAP studies projected from 2020-2040 in the UK. The number of cumulative new CT studies and associated storage required over this period is displayed in the bottom table. Projected

figures assume all files are stored indefinitely and on premise. Emissions were calculated based on the mean file sizes of Cohort B (n=30; a subset of Cohort A), the percentage of studies with each scan type in Cohort A (n=183), and associated total energy consumption (internal storage, server, network, and infrastructure). Cancer incidence statistics were derived from GLOBALCAN (4,5).

MTCO2e = Metric tonnes CO2 equivalent; CT-CAP = CT chest abdomen and pelvis; UK = United Kingdom

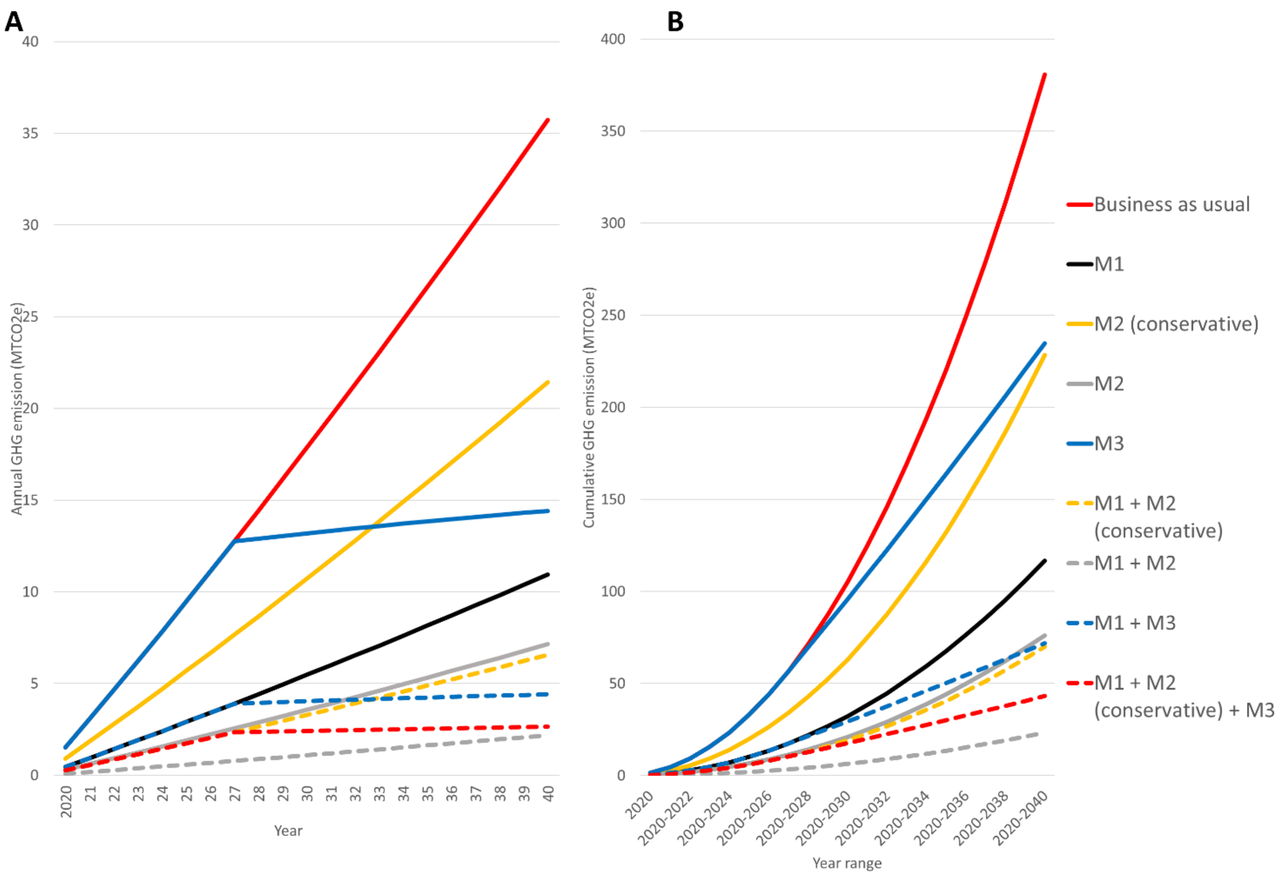

M1 = axial-only storage **M2 = switch to cloud assuming 40% (conservative) or 80% mitigation** **M3 = 8-year retention**

Year range	2020	2020-2022	2020-2024	2020-2026	2020-2028	2020-2030	2020-2032	2020-2034	2020-2036	2020-2038	2020-2040
**Cumulative new CT studies**	11385	34556	58263	82497	107235	132477	158180	184329	210908	237866	265203
**Cumulative stored CT studies (M3)**	11385	34556	58263	82497	96419	99648	102830	105957	109035	112013	114932

Supplementary Figure 2. Greenhouse gas emission associated with the storage of new endometrial cancer staging CT-CAP studies projected from 2020-2040 in the UK with various mitigation strategies. Displayed as annual carbon emission (4A) and cumulative carbon emission (4B). Business as usual assumes all files are stored according to current file sizes and proportions, on-premise, and indefinitely. Mitigation 1 (M1): Storage of only the acquired axial series. M2 (conservative): Switch to cloud storage based on: conservative mitigation of 40%. M2: Switch to cloud storage based on published mitigation of 80% (supplementary table 1). M3: Simplified data retention policy of moving to a permanent archive (with negligible electricity consumption) 8 years after acquisition. The number of cumulative new studies and cumulative stored studies (if M3 was implemented) over this period is displayed in the bottom table. Emissions were calculated based on the mean file sizes of Cohort B (n=30; a subset of Cohort A), the percentage of studies with each scan type in Cohort A (n=183), and associated total energy consumption (internal storage, server, network, and infrastructure). Cancer incidence statistics were derived from GLOBALCAN (4,5).

MTCO2e = Metric tonnes CO2 equivalent; CT-CAP = CT chest abdomen and pelvis; UK = United Kingdom

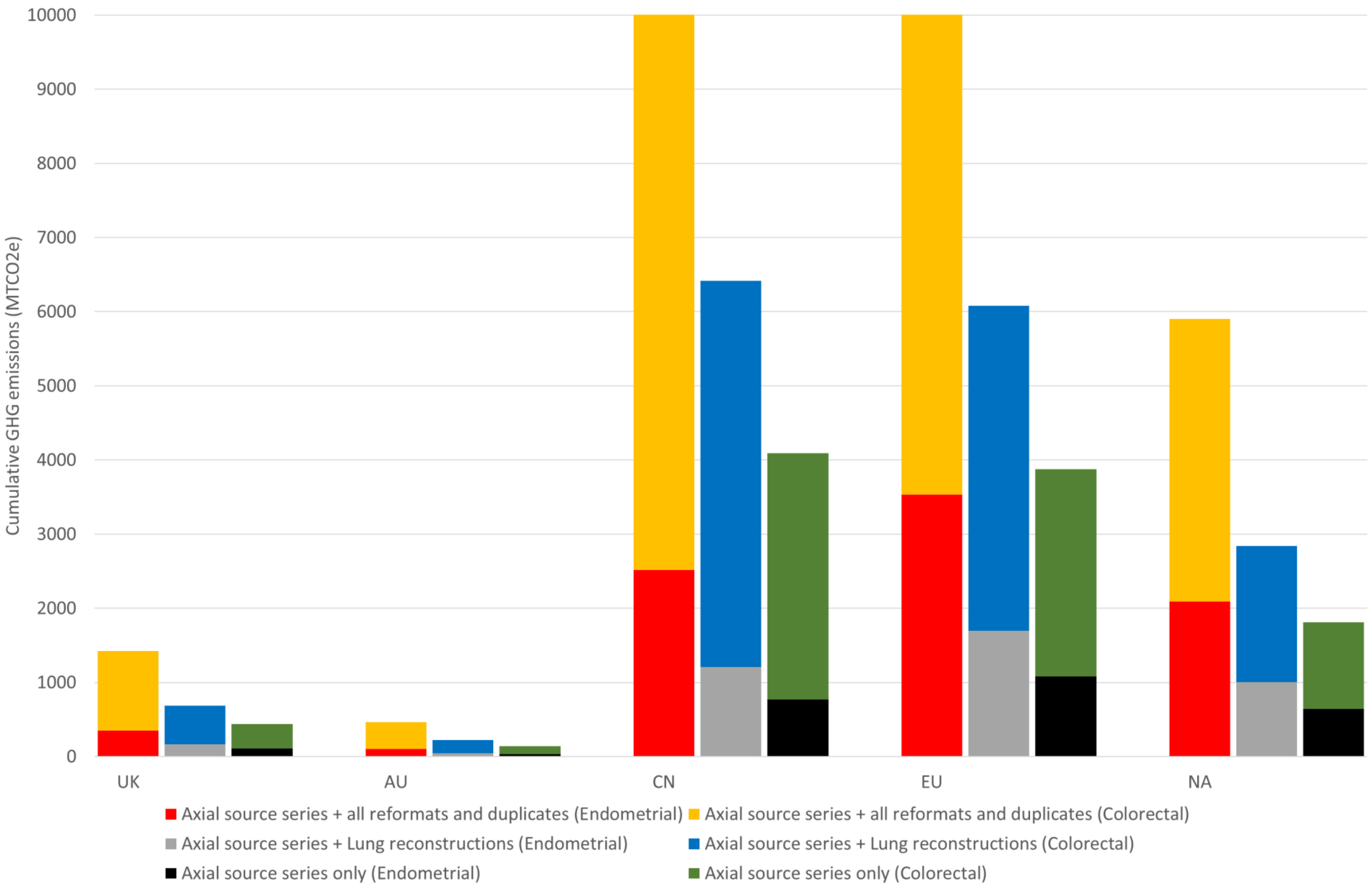

Eur Radiol (2025) Jia Y, Deng M, Burger R, et al.

Supplementary Figure 3. Cumulative greenhouse gas emission associated with on premise storage of new endometrial and colorectal cancer staging CTCAP studies projected from 2020-2040, across each of the five regions (United Kingdom, Australia, China, Europe, North America). Storage-associated emissions due to endometrial cancer are displayed at the bottom; those due to colorectal cancer are displayed at the top. Carbon emission figures assume all files are stored indefinitely and on premise. Emissions were calculated based on the mean file sizes of Cohort B (n=30; a subset of Cohort A), the percentage of studies with each scan type in Cohort A (n=183), associated total energy consumption (internal storage, server, network, and infrastructure), and the emission conversion factor for each region (2). Cancer incidence statistics were derived from GLOBALCAN (4,5).

MTCO2e = Metric tons CO2 equivalent; CT-CAP = CT chest abdomen and pelvis

Region	Number of new cases		Acquired axial series + all reformats and duplicates			Acquired axial series + Lung reconstructions			Acquired axial series only		
	2020	Projected cumulative 2020-2040	On premise storage (MTCO2e)	Cloud storage (MTCO2e (%decrease))		On premise storage (MTCO2e (%decrease))	Cloud storage (MTCO2e (%decrease))		On premise storage (MTCO2e (%decrease))	Cloud storage (MTCO2e (%decrease))	
**UK**	33815	831159	1172	703 (40%)	234 (80%)	563 (52%)	338 (71%)	113 (90%)	359 (69%)	215 (82%)	72 (94%)
**AU**	10861	281532	1045	627 (40%)	209 (80%)	502 (52%)	301 (71%)	100 (90%)	320 (69%)	192 (82%)	64 (94%)
**CN**	306078	8472128	31340	18804 (40%)	6268 (80%)	15071 (52%)	9043 (71%)	3014 (90%)	9606 (69%)	5764 (82%)	1921 (94%)
**EU**	291260	6761470	13384	8030 (40%)	2677 (80%)	6436 (52%)	3862 (71%)	1287 (90%)	4102 (69%)	2461 (82%)	820 (94%)
**NA**	117339	2878123	7132	4279 (40%)	1426 (80%)	3430 (52%)	2058 (71%)	686 (90%)	2186 (69%)	1312 (82%)	437 (94%)
**Total**	725538	18393253	52901	31741 (40%)	10580 (80%)	25439 (52%)	15263 (71%)	5088 (90%)	16214 (69%)	9728 (82%)	3243 (94%)

Supplementary Table 4. Cumulative greenhouse emission associated with the storage of new colorectal cancer staging CTCAP studies projected from 2020-2040, with mitigation by reducing reformat storage and migration to cloud storage.

Projected figures assume all files are stored indefinitely. Projected figures for cloud storage assumes a 40% reduction in GHG as a conservative estimate, or 80% reduction in line with recent reports (supplementary table 1). The % decrease represents GHG savings relative to if acquired axial series + all reformats and duplicates were stored indefinitely on premise. Emissions were calculated based on the mean file sizes of Cohort B (n=30; a subset of Cohort A), the percentage of studies with each scan type in Cohort A (n=183), associated total energy consumption (internal storage, server, network, and infrastructure), and the emission conversion factor for each region (2). Cancer incidence statistics were derived from GLOBALCAN (4,5). All numbers are displayed to zero decimal points.

MTCO_2e = Metric tonnes CO2 equivalent; CTCAP = CT Chest abdomen and pelvis; UK = United Kingdom; AU = Australia; CN = China; EU = European Union; NA = North America

Region	Number of new cases		Acquired axial series + all reformats and duplicates		Acquired axial series + Lung reconstructions		Acquired axial series only	
	**2020**	**Projected cumulative 2020-2040**	No image retention policy (MTCO2e)	Deep storage after eight years (MTCO2e (%decrease))	No image retention policy (MTCO2e (%decrease))	Deep storage after eight years (MTCO2e (%decrease))	No image retention policy (MTCO2e (%decrease))	Deep storage after eight years (MTCO2e (%decrease))
**UK**	33815	831159	1172	729 (38%)	563 (52%)	351 (70%)	359 (69%)	223 (81%)
**AU**	10861	281532	1045	659 (37%)	502 (52%)	317 (70%)	320 (69%)	202 (81%)
**CN**	306078	8472128	31340	19964 (36%)	15071 (52%)	9600 (69%)	9606 (69%)	6119 (80%)
**EU**	291260	6761470	13384	8292 (38%)	6436 (52%)	3988 (70%)	4102 (69%)	2542 (81%)
**NA**	117339	2878123	7132	4459 (37%)	3430 (52%)	2144 (70%)	2186 (69%)	1367 (81%)
**Total**	725538	18393253	52901	33374 (37%)	25439 (52%)	16049 (70%)	16214 (69%)	10230 (81%)

Supplementary Table 5. Cumulative greenhouse gas emission associated with the storage of new colorectal cancer staging CTCAP studies projected from 2020-2040, with mitigation by reducing reformat storage and simplified data retention strategy.

All figures assume storage on premise. Projected figures for No image retention policy assume all files are stored indefinitely. Projected figures for Deep storage after eight years assume negligible electricity consumption in deep storage using hard drives. The % decrease represents carbon savings relative to if axial source series + all reformats and duplicates were stored indefinitely. Emissions were calculated based on the mean file sizes of Cohort B (n=30; a subset of Cohort A), the percentage of studies with each scan type in Cohort A (n=183), associated total energy consumption (internal storage, server, network, and infrastructure), and the emission conversion factor for each region (2). Cancer incidence statistics were derived from GLOBALCAN (4,5). All numbers are displayed to zero decimal points.

MTCO$_2$e = Metric tonnes CO2 equivalent; CTCAP = CT Chest abdomen and pelvis; UK = United Kingdom; AU = Australia; CN = China; EU = European Union; NA = North America

Region	Number of new cases				
	**2020**	**Projected cumulative 2020-2040**	**Business as usual** (MTCO2e)	**Axial source series + Lung reconstructions + Cloud migration (Conservative) + Deep storage after eight years** (MTCO2e (%decrease))	**Axial source series + Lung reconstructions + Cloud migration (Conservative) + Deep storage after eight years** (MTCO2e (%decrease))
**UK**	11385	265203	381	68 (82%)	43 (89%)
**AU**	3055	76430	281	50 (82%)	32 (88%)
**CN**	81964	1888388	7275	1289 (82%)	822 (89%)
**EU**	188583	2634564	5186	915 (82%)	583 (89%)
**NA**	68378	1579239	3912	694 (82%)	443 (89%)
**Total**	271980	6178621	16654	2949 (82%)	1880 (89%)

Supplementary Table 6. Cumulative greenhouse gas emission associated with the storage of new endometrial cancer staging CTCAP studies projected from 2020-2040, with combined mitigation strategies.

Business as usual assumes all files (axial source + all post-processed series) are stored indefinitely, on-premise. Projected figures for cloud storage assumes a 40% reduction in GHG as a conservative estimate. Projected figures for Deep storage after eight years assume negligible electricity consumption in deep storage using hard drives. The % decrease represents carbon savings relative to business as usual. Emissions were calculated based on the mean file sizes of Cohort B (n=30; a subset of Cohort A), the percentage of studies with each scan type in Cohort A (n=183), associated total energy consumption (internal storage, server, network, and infrastructure), and the emission conversion factor for each region (2). Cancer incidence statistics were derived from GLOBALCAN (4,5). All numbers are displayed to zero decimal points.

$MTCO_2e$ = Metric tonnes CO2 equivalent; CTCAP = CT Chest abdomen and pelvis; UK = United Kingdom; AU = Australia; CN = China; EU = European Union; NA = North America

Region	Number of new cases				
	**2020**	**Projected cumulative 2020-2040**	**Business as usual** (MTCO2e)	**Axial source series + Lung reconstructions + Cloud migration (Conservative) + 8yr retention** (MTCO2e (%decrease))	**Axial source series + Lung reconstructions + Cloud migration (Conservative) + 8yr retention** (MTCO2e (%decrease))
**UK**	33815	831159	1172	563 (52%)	351 (70%)
**AU**	10861	281532	1045	502 (52%)	317 (70%)
**CN**	306078	8472128	31340	15071 (52%)	9600 (69%)
**EU**	291260	6761470	13384	6436 (52%)	3988 (70%)
**NA**	117339	2878123	7132	3430 (52%)	2144 (70%)
**Total**	725538	18393253	52901	25439 (52%)	16049 (70%)

Supplementary Table 7. Cumulative greenhouse gas emission associated with the storage of new colorectal cancer staging CTCAP studies projected from 2020-2040, with combined mitigation strategies.

Business as usual assumes all files (axial source + all post-processed series) are stored indefinitely, on-premise. Projected figures for cloud storage assumes a 40% reduction in GHG as a conservative estimate. Projected figures for Deep storage after eight years assume negligible electricity consumption in deep storage using hard drives. The % decrease represents carbon savings relative to business as usual. Emissions were calculated based on the mean file sizes of Cohort B (n=30; a subset of Cohort A), the percentage of studies with each scan type in Cohort A (n=183), associated total energy consumption (internal storage, server, network, and infrastructure), and the emission conversion factor for each region (2). Cancer incidence statistics were derived from GLOBALCAN (4,5). All numbers are displayed to zero decimal points.

$MTCO_2e$ = Metric tonnes CO2 equivalent; CTCAP = CT Chest abdomen and pelvis; UK = United Kingdom; AU = Australia; CN = China; EU = European Union; NA = North America

References

1. Department for Business, Energy & Industrial Strategy (BEIS). Energy Follow Up Survey 2017: Household Energy Consumption & Affordability - Final Report [Internet]. London; 2021 [cited 2023 Dec 12]. Available from: https://assets.publishing.service.gov.uk/media/61449c45e90e07044435c9e7/efus-Household-Energy-Consumption-Affordability.pdf#page=5&zoom=100,57,310

2. Ember Energy. Yearly Electricity Data [Internet]. 2024 [cited 2025 May 18]. Available from: https://ember-energy.org/data/yearly-electricity-data

3. United Nations, Department of Economic and Social Affairs, Population Division. World Population Prospects 2024 [Internet]. 2024 [cited 2025 May 18]. Available from: https://population.un.org/wpp/

4. International Agency for Research on Cancer. Global Cancer Observatory: Cancer Today [Internet]. [cited 2023 Dec 14]. Available from: https://gco.iarc.fr/today

5. International Agency for Research on Cancer. Global Cancer Observatory: Cancer Tomorrow [Internet]. [cited 2024 Aug 18]. Available from: https://gco.iarc.who.int/today/
